# Draft Genome Sequences of Six Novel Bacterial Isolates from Chicken Ceca

**DOI:** 10.1128/genomeA.00448-16

**Published:** 2016-05-26

**Authors:** Nicholas A. Duggett, Gemma L. Kay, Martin J. Sergeant, Michael Bedford, Chrystala I. Constantinidou, Charles W. Penn, Andrew D. Millard, Mark J. Pallen

**Affiliations:** a Microbiology and Infection Unit, Warwick Medical School, University of Warwick, Coventry, United Kingdom; b School of Biosciences, University of Birmingham, Birmingham, United Kingdom; c AB Vista Feed Ingredients, Marlborough, United Kingdom

## Abstract

The chicken is the most common domesticated animal and the most abundant bird in the world. However, the chicken gut is home to many previously uncharacterized bacterial taxa. Here, we report draft genome sequences from six bacterial isolates from chicken ceca, all of which fall outside any named species.

## GENOME ANNOUNCEMENT

We cultured 37 bacterial isolates from post-mortem cecal contents of commercially raised 35-day-old Ross broilers. 16S rRNA gene sequencing suggested that six isolates were distinct from all previously named bacterial species due to placement in a taxonomic tree built with ARB, and so these isolates were selected for whole-genome sequencing (1). Three rounds of colony purification were carried out before genomic DNA extraction using a modified Qiagen stool extraction kit. Genomic DNA (1 ng) was prepared using the Nextera XT DNA sample preparation kit (Illumina) followed by sequencing on the Illumina MiSeq platform using the paired-end 2 × 250-bp (version 2) protocol. The resultant reads were checked for quality with fastqc (version 0.11.3 [http://www.bioinformatics.babraham.ac.uk/projects/fastqc]) and trimmed with Sickle (version 1.33 [https://github.com/najoshi/sickle]). *De novo* genome assembly was performed using SPAdes3.1 (2). To check for errors, reads were mapped against the assembly using BWA MEM (3). After mapping, contigs with <5× coverage were excluded and any errors in base calling corrected. All genomes were sequenced to a minimum coverage of 24×, with a median coverage of 41× across all isolates. Contigs were annotated with Prokka1.11 (4).

The genomes varied in size from 2.49 Mb/2248 coding sequences (CDSs) for CHCKI005 to 3.99 Mb/3686 CDSs for CHCKI004. All isolates harbor between one (CHKCI002) and six (CHKCI006) putative prophages. Using 40 single copy phylogenetic marker genes (5), none of the isolates could be classified at the species level. An *in silico* DNA-DNA hybridization analysis of the genomes was completed using GGDC2.0 (6) and average nucleotide identity was performed using JSpecies (7) against the nearest bacterial species (determined by placement in ARB) to ensure the isolates did not belong to known species. 16S rRNA gene sequences were analyzed using the ARB software package and the LTP_121 database from the All-Species Living-Tree project. Sequences were aligned with SINA aligner within ARB and inserted into the tree using the ARB parsimony method. Isolates could be classified at various taxonomic levels: isolate CHKCI003 represents a new species within the genus *Alistipes*, isolate CHKCI001 falls within the family *Lachnospiraceae*, isolate CHKCI002 falls within the family *Coriobacteriaceae*, and isolates CHKCI004, CHKCI005, and CHCKI006 represent previously uncharacterized species within the order *Clostridiales*.

### Nucleotide sequence accession numbers.

The draft genome sequences of isolates CHKCI001, CHKCI002, CHKCI003, CHKCI004, CHKCI005, and CHKCI006 have been deposited in DDBJ/ENA/GenBank under the accession numbers FCNS01000001, FCNB01000001, FCNT01000001, FCNR01000001, FJVJ01000001, and FCNA01000001, respectively.
